# Novel Palladium(II) Complexes that Influence Prominin-1/CD133 Expression and Stem Cell Factor Release in Tumor Cells

**DOI:** 10.3390/molecules22040561

**Published:** 2017-03-30

**Authors:** Eva Fischer-Fodor, Roman Mikláš, Lucia Rišiaňová, Mihai Cenariu, Ioana Georgeta Grosu, Piroska Virag, Maria Perde-Schrepler, Ciprian Tomuleasa, Ioana Berindan-Neagoe, Ferdinand Devínsky, Natalia Miklášová

**Affiliations:** 1 “I. Chiricuta” Institute of Oncology, Republicii 34-36, RO-400015 Cluj-Napoca, Romania; fischer.eva@iocn.ro (E.F.-F.); virag.piroska@yahoo.com (P.V.); pmariaida@yahoo.com (M.P.S.); ciprian.tomuleasa@umfcluj.ro (C.T.); Ioana.neagoe@umfcluj.ro (I.B.N.); 2 Medfuture Research Center for Advanced Medicine “Iuliu Hatieganu”, University of Medicine and Pharmacy, Babes 8, RO-400012 Cluj-Napoca, Romania; 3 Department of Chemical Theory of Drugs, Faculty of Pharmacy, Comenius University in Bratislava, Kalinčiakova 8, 83104 Bratislava, Slovakia; miklas@fpharm.uniba.sk (R.M.); lucia.risianova@gmail.com (L.R.); devinsky@fpharm.uniba.sk (F.D.); 4 Biotechnology Research Center, University of Agricultural Science and Veterinary Medicine, 400372 Cluj-Napoca, Romania; Mihai.cenariu@usamvcluj.ro; 5 National Institute for Research and Development of Isotopic and Molecular Technologies, RO-400293 Cluj-Napoca, Romania; grosu.ioana@gmail.com

**Keywords:** palladium complexes, curcuminoids, colorectal cancer, prominin-1 expression, cytotoxicity, stem cell factor

## Abstract

New Pd(II) complexes of 1,7-bis(2-methoxyphenyl)hepta-1,6-diene-3,5-dione were synthesized and structurally characterized. The complexes were tested in vitro on human colon and hepatic carcinoma cell lines, normal hepatic cells and hematopoietic progenitor cells. Biological tests proved that Pd(II) complexes **1** and **2** (containing a curcumin derivative) exhibit a strong in vitro antitumor effect against the cells derived from human colorectal carcinoma and the hepatic metastasis of a colorectal carcinoma. Complex **1** has an outstanding inhibitory effect against BRAF-mutant colon carcinoma and hepatocarcinoma cell growth; **1** and **2** are both more active than the free ligand and have the capacity to trigger early apoptotic processes. By flow cytometric measurements, an important decrease of prominin-1 (CD133) molecule expression on tumor cells membrane was identified in cell populations subjected to **1** and **2**. Quantitative immune enzymatic assay proved restrictions in stem cell factor (SCF) release by treated tumor cells. Although less cytotoxic, the free ligand inhibits the surface marker CD133 expression in hepatocarcinoma cells, and in HT-29 colon carcinoma. The new synthesized Pd(II) complexes **1** and **2** exhibit an important potential through their selective cytotoxic activity and by targeting the stem-like tumor cell populations, which leads to the tumor growth arrest and prevention of metastasis.

## 1. Introduction

In the last decades, modern cancer treatment modalities have extended the survival of patients with solid tumors, but the mortality rate remains unchanged [1]. Treatment failure and multidrug resistance are common in colorectal cancers, the third most commonly diagnosed cancer in males and second in the female population [2]. Recent studies have described the major importance of cancer stem-like cells within a tumor; these cells drive the tumor growth and evade therapy [3] through asymmetric division, self-renewal and differentiation ability, therefore the evidence of these cells required improvements in standard cancer treatment [4]. Preferably, the anti-cancer drugs and other treatment methods should eliminate not only the rapidly dividing and terminally differentiated cell populations, but they should target the dormant stem-like cancer cells, which confer chemoresistance. CD133 (or Prominin-1) is an important stem cell marker in colorectal carcinoma associated with tumorigenicity and progression of the disease. The up-regulation of CD133 in colorectal cancer strongly correlates with a poor prognosis [5]. It is presumed that a targeted therapy which focuses on CD133, prevents the tumor re-growth and metastasis [6] rather than shrinkage of tumor size in short term. Curcumin, the natural product extracted from *Curcuma longa* rhizome, has a wide usage in medicine, food industry and cosmetics, based on its beneficial properties. This biologically active component proved antioxidant, anti-inflammatory, antitumor activities and it was found to be useful in many chronic diseases, including cancer [7,8]. The attempt to bring curcumin into antitumor chemotherapy protocols together with standard drugs led to the reduction of colon stem-like cancer cells in vitro [9]. Although, curcumin and its analogues manifest a noticeable biological activity, they exhibit poor bioavailability because of low absorption, rapid metabolism, and rapid systemic elimination [10], having a limited solubility in water and other solvents. Several curcumin analogues including 1,7-bis(2-methoxyphenyl)hepta-1,6-diene-3,5-dione, displayed antioxidant activity [11], suppression of the NF-κB expression through the tumor necrosis factor-α pathway [12] and anti-inflammatory activity [13,14]. Derivatives of curcumin with appropriate substituents in the 4th position played an important role in the chemoprevention and chemotherapy of glioma and skin cancer [15]. Moreover, halogenated curcumin analogues having the ability to bind vitamin D receptor, may low the risk of colon and epithelial cancer [16]. On the other hand, in cancer chemotherapy protocols the metal-based drugs have gained an important role, therefore curcumin and its metal complexes were intensely studied for their therapeutic properties, including the gastrointestinal cancers [8,17]. Although, the oxaliplatin drug is by now a choice in colorectal cancer treatment [18], recently palladium was extensively tested also, in the form of coordinative compounds with biologically active ligands, in vitro, in colon cancer [19,20,21]. Although platinum and palladium complexes are commonly used in the cancer therapy [22], those containing curcumin or curcumin’s analogues also proved to be effective as antitumor agents [23,24,25,26,27]. In former studies, we had the confirmation of efficiency of metal complexes of curcumin concerning the antineoplastic activity in ovarian, colorectal, melanoma, cervical, liver and breast carcinomas [28,29,30], a fact that encouraged us to further investigations of such coordination models. Curcumin acts against cancer stem cells by interferences with several signaling pathways [31], and the coordination of curcumin and its analogues to metals may increase the selectivity for biological targets and improve their bioavailability levels in tumor cells [17]. Herein, the synthesis, characterization and biological application as antitumor biomaterials of Pd(II) complexes with 1,7-bis(2-methoxyphenyl)hepta-1,6-diene-3,5-dione are described. The new palladium(II) complexes (**1** and **2**) growth inhibition was assessed in vitro on human colorectal (HT-29 and DLD-1) cell populations and hepatic CSC stem-like tumor cells derived from a hepatic metastasis. To emphasize their selectivity, identical assessments were made on normal liver cells (LIV) and on normal progenitor hematopoietic blood cells. The mechanism of action of complexes **1**, **2** and of curcumin-like ligand was elucidated tracking an important stem cell marker: the prominin-1 or CD133 expression of the treated cells membrane. Moreover, Stem Cell Factor (SCF) release was also measured in vitro. The biologic outcome of the novel complexes indicates that they are better prodrugs as the free ligand, and proved the Pd(II) complexes capacity to target the cancer stem-like cells which sustain the tumor growth.

## 2. Results and Discussion

### 2.1. Synthesis and Characterization

**Scheme 1:**
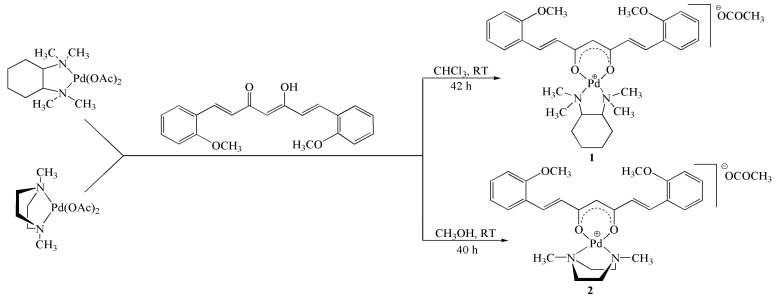
Synthesis of palladium(II) complexes **1** and **2**.

Two palladium(II) complexes with 1,7-bis(2-methoxyphenyl)hepta-1,6-diene-3,5-dione were synthesized and structurally characterized. Coordination of the free ligand with palladium was meant for improving the compounds bioavailability and toxicity by increasing its selectivity and targeting the tumor cells. Novel Pd(II) complexes **1** and **2**, were obtained by reacting the precursor palladium complexes [(C_10_H_22_N_2_)Pd(OAc)_2_] and [(C_6_H_14_N_2_)Pd(OAc)_2_] with the curcumin analogue, 1,7-bis(2-methoxyphenyl)hepta-1,6-diene-3,5-dione in an equimolecular ratio (Scheme 1). All complexes, including also the precursors have been characterized by ^1^H- and ^13^C-NMR spectroscopy, IR spectroscopy, mass spectrometry, the analytical data being presented in the Materials and Methods and Supplementary Material sections.

### 2.2. Cytotoxicity Assessments

**Figure 1:**
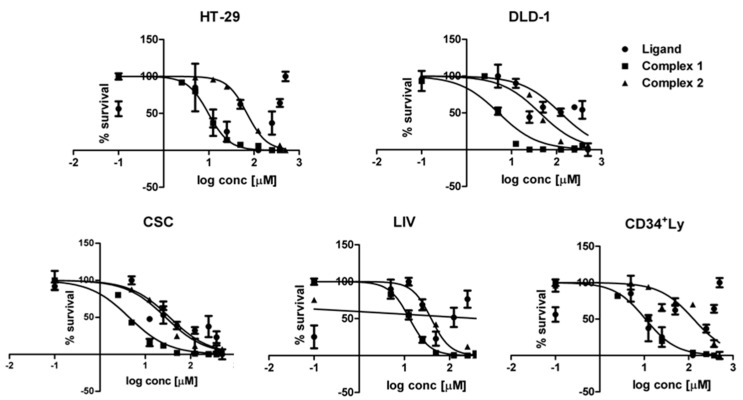
Dose-response sigmoidal curves corresponding to the treatment of tumor and normal cells in vitro with the free ligand and with complexes **1** and **2**.

In vitro, the Pd(II)complexes exhibit a growth inhibitory effect against the colon, hepatic and hematopoietic cells; half inhibitory concentrations (IC_50_) were calculated using the sigmoid dose-response curves (Figure 1).

The growth inhibitory effect of the complexes **1** and **2** is several fold higher as of 1,7-bis(2-methoxyphenyl)hepta-1,6-diene-3,5-dione; a low IC_50_ indicates a superior cytotoxicity (Table 1). The one-way analysis of variance and the Dunnet post-test indicates a very significant difference between the antiproliferative activity of ligand vs. Pd(II) complexes in HT-29, DLD-1 and LIV cell lines (*p* < 0.01). A significant effect is observed also in CSC and CD34 positive lymphocyte populations (*p* < 0.05). In every cell line, complex **1** effect is prominent (*p* < 0.05), being the most active against all the cell lines (Table 1, Figure 1).

Paradoxically, complexes **1** and **2** and the curcumin analogue exhibit the best effect against the CSC cell line (Figure 1), which display the highest proliferation rate among the studied cell populations. Since the cells turnover is more rapid in this cancer stem-like cell line, the compounds **1**, **2** and the ligand might inhibit in the first 24 h of treatment the molecules of β-catenin, interleukins and transmembrane proteins [32], which control the stem cells’ proliferation. The effect of complex **1** is slightly reduced in normal LIV hepatic cells and lymphocytes in comparison with the colon tumor cells and with CSC liver stem-like tumor cells (one-way Anova test, *p* < 0.05), but it is still toxic against all cell types. Complex **1** exhibits the best selectivity towards the cancer cells, while in the case of complex **2**, the antiproliferative activity is almost similar in normal LIV and malignant DLD-1 cells. Moreover, complex **2** displays a lower effect against the HT-29 cells as compared with the normal hepatic cells. The treatment with 1,7-bis(2-methoxyphenyl)hepta-1,6-diene-3,5-dione and with complexes **1** and **2** has influence on CD34 positive cells population viability. The ligand has a low effect and the toxicity of **1** follows the same trend as in tumor cells and normal hepatocytes. Complex **2** shows a significantly lower toxicity on cluster of differentiation (CD34) positive hematopoietic stem cells. In colon cancer CD34 antigen is a marker of the microvessel density [33], being expressed on endothelial cells which contribute to the neoangiogenesis in the malignant tissues, therefore the compound has a good potential to interact with the tumor microenvironment as well.

### 2.3. Prominin-1 (CD133) Expression

Prominin-1 or CD133 is expressed as membrane marker on normal and tumor stem cells, and in many colorectal [34] and hepatic cell lines [35], and its expression influences the balance between survival and apoptosis of these malignant cells [36].

**Figure 2:**
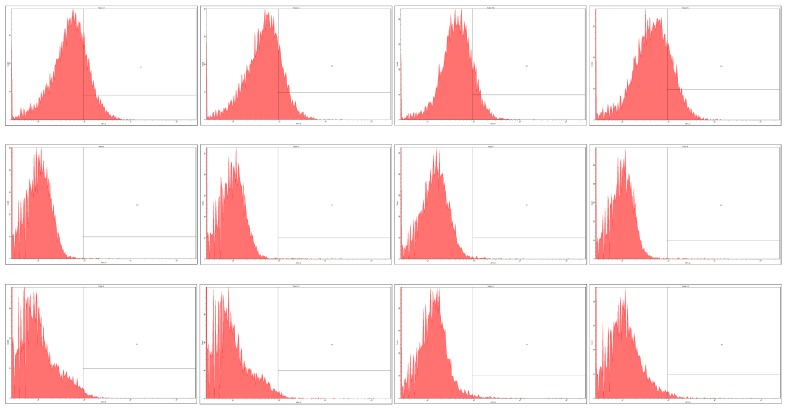
The free ligand and Pd(II) complexes **1** and **2** induced changes of CD133 (prominin-1) expression in the treated tumor cell populations; on x axis the cells fluorescence is depicted (cells marked with allophycocyanin conjugated CD133) and on y axis the cell number was represented. The histograms corresponding to HT-29 (**upper row**), DLD-1 (**middle row**) and CSC hepatic carcinoma stem-like cells (**lower row**) shows differences in the proportion of positive cells and the fluorescence intensity; from left to right: untreated cells, treatment with the free ligand, complex **1** and complex **2**, respectively.

The basal CD133 value differs significantly in each cell line. We have found elevated values of CD133 in HT-29 cell line as described before [5,37] and this was the population with the biggest basal CD133^+^ expression; while in DLD-1 cell line we found the lowest expression among all the studied cell lines (Figure 2, Table 2). The CSC malignant liver cell population is richest in CD133-positive cells as the normal LIV cell line. In HT-29 and CSC cells, all compounds diminished the CD133 proportion, significant being only complex **1** (Figure 2). The free ligand effect has a statistical significance only in HT-29 cells, and complex **2** influences positively the growth in CSC. In DLD-1 population none of the tested compounds reduces the fraction of CD133 positive cells (one-way analysis of variance, Bonferroni multiple comparison test in the 95% confidence interval).

**Figure 3:**
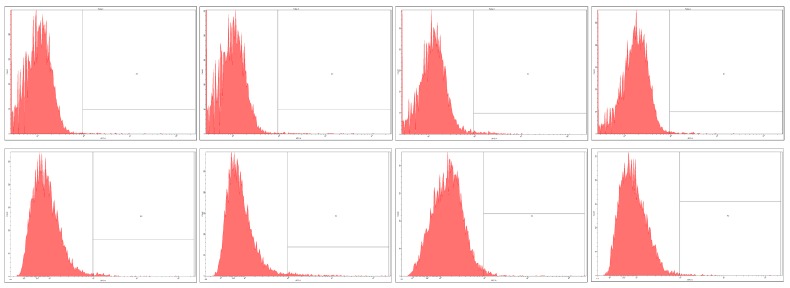
Modulation of CD133/prominin-1 expression by curcumin-like ligand and its complexes **1** and **2** in normal human cells: LIV normal hepatic stem-like cells (**upper row**) and hematopoietic progenitor CD34-positive lymphocytes (**lower row**). From left to right: untreated cells, cells treated with ligand, with complex **1** and complex **2**. The fluorescence intensity given by the allophycocyanine-CD133 marked cells is represented on x axis, and the positive cells number is depicted on the y axis.

An important aspect is the behaviour of normal LIV cells, where the curcumin analogue and the complexes **1** and **2** increased the CD133-positive cells proportion after a 24 h treatment, proving that the normal stem-like cells are not affected (Figure 3). In the CD34 positive hematopoietic stem cells the expression of CD133 increases following the treatment with the ligand and with the complex **1**, while complex **2** significantly decreases the proportion of double positive CD133^+^CD34^+^ cells.

CD133 is a transmembrane glycoprotein with an extracellular N-terminus, a cytoplasmic C-terminus and cysteine rich cytoplasmic and extracellular loops [38]. In metal-functionalized nanostructures it was evidenced that palladium bind to glycoproteins C-terminus [39], therefore we can’t exclude a direct interaction of **1** and **2** with CD133. Pd can exert as well an indirect influence on CD133 through the phosphatidylinositol 3-kinase PI3K/Akt signaling pathway. **1** or **2** has a weaker effect on DLD-1 cells, since the overexpression of CD133 is linked to K-ras gene mutation, a characteristic of these cells.

In vitro tests proved that curcumin derivatives manifest the capacity to reduce CD133 membrane marker expression and reduce the CD133 positive stem cells proliferation by counteracting the epithelial-mesenchymal transition in a tridimensional tumor model [40]. However, the reduced bioavailability of curcumin, which target many other cell types not only the cancer cells [10], could be an impediment to obtain the same results in vivo in living organisms. Therefore, by chemical derivatization of the curcuminoid ligand our aim was to target the highly proliferative tumor cells.

CD133 up-regulation in colorectal cancers is associated with progression of the disease, poor prognosis and drug resistance [5,8], therefore the decrease of the stem-like tumoral CD133^+^ cells proportion in HT-29 colon carcinoma and CSC hepatic metastasis due to exposure to complex **1** indicates good therapeutic perspectives, especially because the compound simultaneously enhances the expression of CD133/prominin-1 of normal stem cells.

### 2.4. Stem Cell Factor

The soluble form of SCF in human serum is associated with the level of malignancy, suggesting that it could serve as a tumor marker [41] in colorectal cancer. The overexpression of SCF enhances the cellular proliferation and invasion in the colorectal cancer by binding and activating the receptor tyrosine kinase c-Kit through the PI3K/Akt signaling pathway, therefore the SCF/cKit system became an appropriate target for cancer therapies [42].

**Figure 4:**
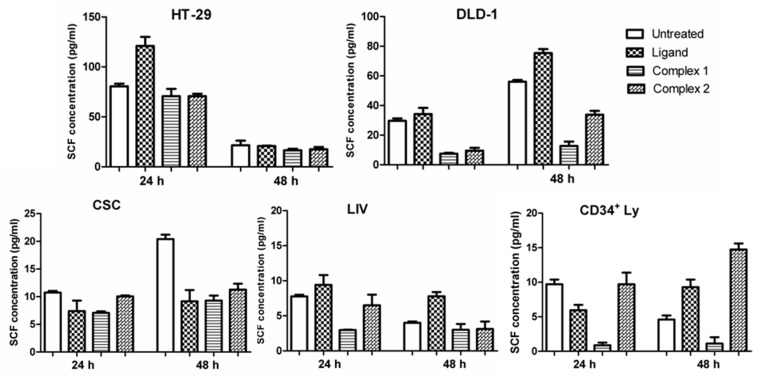
The level of soluble SCF secreted by malignant and normal cell lines in vitro is modulated by ligand and Pd(II) complexes **1** and **2** treatment.

The SCF secretions of the treated cells were estimated with a quantitative immunoenzymatic method, enzyme-linked immunosorbent assay (ELISA) after 24 h of treatment. However, the lasting effect of compounds for the next 24 h after removing them from the cell cultures, was taken into consideration too (Figure 4).

The level of SCF varies in the cell cultures media depending on the cell type: in the normal stem-like liver LIV cell line and in human CD34 positive progenitor lymphocytes primary culture, the basal SCF expression is significantly lower as in DLD-1 and HT-29 colon carcinoma cells. No significant differences between the normal cell lines and the CSC hepatic stem-like tumor cells can be observed. In untreated DLD-1 and CSC tumor cell populations the stem cell factor values in the first 24 h are usually lower as in the next 24 h, only in HT-29 the SCF values are much more elevated in the first 24 h of treatment. In normal LIV cells and CD34 positive lymphocytes the tendency is opposite as in tumor cells. In cell culture supernatants, lower values as reported in human serum plasma [43] were found. The cells exposure to curcumin analogue does not suppress the SCF secretion in vitro, contrary, in HT-29 and DLD-1 carcinoma cells and LIV normal cells the SCF level rises after 24 h treatment and continues to elevate in the next 24 h too. Only in CSC hepatic tumor cells the ligand diminishes SCF. In normal hematopoietic CD34^+^ stem cells the inhibitory effect of the ligand ceases when the exposure to compounds is ended.

Compound **1** inhibits very significantly the SCF production in DLD-1 after 24 h of treatment and 24 h after the treatments end, but in HT-29 cells does not display the same effect. In CSC tumor cells, the inhibitory effect was not evident after 24 h, but after 48 h the SCF level reduction was statistically significant. In LIV the changes in SCF are not significant, while in CD34^+^ hematopoietic cell populations the decrease of SCF level after 24 h of treatment is promptly restored (one-way analysis of variance, Turkey post-test, *p* < 0.05).

Compound **2** inhibits the SCF production in DLD-1 after 24 h of treatment and the decrease maintains at 48 h as well. In CSC even if no significant effect was detected after 24 h, the cessation of the treatment corresponded to the SCF concentration decrease (one-way analysis of variance, Turkey post-test, *p* < 0.05). In HT-29 cells no significant decrease was observed at 24 h or 48 h checkpoints. As regards the normal cell behavior, following the treatment the SCF secretion does not modify significantly in LIV at any time point. In CD34 positive hematopoietic stem cells during the treatment was no significant change and after cessation of the treatment, SCF secretion was enhanced, not inhibited. Human colorectal carcinoma cells: HT-29 and DLD-1 co-express and overexpress the receptor tyrosine kinase c-Kit and its ligand SCF and the increase of SCF level in their cell growth medium contributes to anchorage-independent expansion [44]. Hundreds of protein phosphorylation sites were identified in HT-29 human colon adenocarcinoma cell line which display a different SCF pattern, unlike the other studied cell lines: DLD-1, CSC, LIV and the hematopoietic stem cells. The HT-29 cell line exhibit the BRAF mutation and not the K-ras mutation like DLD-1, which could influence through several chemokines the c-Kit-SCF ligand formation, and the drug targeting can be activated by SCF. KIT is expressed only in DLD-1 cells and this will influence the phosphorylation and the soluble SCF level following the treatment. **1** exhibits a good selectivity towards DLD-1 more likely because the diammine palladium moiety of **1** acts against the KIT receptor, while dimethylpiperazine group of **2** influence only SCF.

## 3. Materials and Methods

### 3.1. General Information

All chemicals for syntheses (3,4-dimethoxybenzaldehyde, acetylacetone, B_2_O_3_, tri-*n*-butyl borate, *n*-butylamine, ethylacetate, chloroform, methanol, Pd(OAc)_2_,) were of reagent grade and were used as received. Chloroform was refluxed over CaH_2_ and ethyl acetate was refluxed over CaCO_3_ and then freshly distilled. *N*,*N*,*N*′,*N*′-tetramethylcyclohexane-1,2-diamine [45] and 1,4-dimethylpiperazine [46] were prepared as described previously in the literature. Purification of final products was done by column chromatography performed on silica gel (silica 0.035–0.070 mm 60 Å). The IR spectra were recorded on a 6700 FT-IR spectrophotometer (Nicolet, Waltham, MA, USA) scanning between 500 and 4000 cm^−1^. NMR spectra were measured on a Gemini 2000 spectrometer (Varian, Palo Alto, CA, USA) at working frequencies 300 MHz (for ^1^H-NMR) and 75 MHz (for ^13^C-NMR). Spectra were measured in CD_3_OD, using as internal standard TMS (tetramethylsilane). The chemical shifts (δ) are reported in parts per million (ppm) and the splitting of the protonresonances in ^1^H NMR spectra is defined as follows: s = singlet, d = doublet, t = triplet and m = multiplet. High resolution mass spectra (HRMS) were recorded in positive mode, on a LTQ XL Orbitrap spectrometer (ThermoScientific, San Jose, CA, USA) using the electrospray ionization technique. Melting point was determined with a Koffler hot stage without correction.

### 3.2. Synthesis

Curcumin derivative 1,7-bis(2-methoxyphenyl)hepta-1,6-diene-3,5-dione was prepared according to a known procedure [47]. The precursor palladium complexes with (*R*,*R*)-*N*,*N*,*N*′,*N*′-tetramethylcyclohexane-1,2-diamine[(C_10_H_22_N_2_)Pd(OAc)_2_] and with 1,4-dimethylpiperazine[(C_6_H_14_N_2_)Pd(OAc)_2_] were synthesized based on a procedure reported on our previous studies [29].

#### 3.2.1. Synthesis of Complex **1**

To a solution of 0.15 g (0.45 mmoles) of 1,7-bis(2-methoxyphenyl)hepta-1,6-diene-3,5-dione in dry chloroform (5 mL) were added dropwise precursor complex [(C_10_H_22_N_2_)Pd(OAc)_2_] (0.18 g, 0.45 mmol) dissolved in chloroform (3 mL). Reaction mixture was kept on stirring at room temperature for 42 h. After that, the solvent was removed under vacuum and the final product was purified by silica gel chromatography (CHCl_3_:CH_3_OH, 9:1). The pure product was isolated as a yellowish solid (0.07 g, 23%), m.p. 162–165 °C. ^1^H-NMR (CD_3_OD) δ (ppm) 1.22–1.35 (m, 2H) 1.46–1.58 (m, 2H) 1.82 (d, 1H) 1.88 (s, 3H) 2.22 (d, 1H) 2.85 (s, 6H) 2.87 (s, 6H) 3.22–3.27 (m, 2H) 3.90 (s, 6H) 5.88 (s, 1H) 6.87 (d, 2H) 6.98 (t, 2H) 7.04 (d, 2H) 7.38 (t, 2H) 7.63 (d, 2H) 7.82 (d, 2H). ^13^C-NMR (CD_3_OD) δ (ppm) 24.53 (1C) 25.33 (2C) 25.56 (2C) 43.64 (1C) 43.66 (1C) 56.26 (4C) 73.30 (2C) 106.08 (1C) 112.56 (2C) 122.03 (2C) 125.05 (2C) 126.24 (2C) 129.30 (2C) 132.80 (2C) 136.81 (2C) 159.76 (2C) 180.54 (2C) 180.63 (1C). IR ν_max_ (cm^−1^) 3354, 2948, 1611, 1574, 1507, 1486, 1465, 1395, 1322, 1247, 1180, 1106, 1023, 994, 880, 764, 712, 632, 608. HRMS: Calcd for C_31_H_41_N_2_O_4_Pd [M + H]^+^: 611.2101, found: 611.2097.

#### 3.2.2. Synthesis of Complex **2**

A solution of palladium(II) complex [(C_6_H_14_N_2_)Pd(OAc)_2_] (0.27 g, 0.79 mmoles) in dry methanol (5 mL) was added dropwise to 1,7-bis(2-methoxyphenyl)hepta-1,6-diene-3,5-dione (0.26 g, 0.79 mmol) dissolved in methanol (10 mL). The reaction mixture was stirred at room temperature for 40 h. The final product was purified by silica gel chromatography (CHCl_3_/CH_3_OH 9:1) and 0.09 g (19%) of a pure compound were isolated as an orange solid, m.p. 145–150 °C. ^1^H-NMR (CD_3_OD) δ (ppm) 1.90 (s, 3H) 2.64 (s, 6H) 2.76 (d, 4H) 3.88 (s, 6H) 3.92 (d, 4H) 5.89 (s, 1H) 6.89 (d, 2H) 6.97 (t, 2H) 7.03 (d, 2H) 7.37 (t, 2H) 7.61 (d, 4H) 7.91 (d, 2H). ^13^C-NMR (CD_3_OD) δ (ppm) 23.83 (1C) 46.55 (2C) 56.21 (2C) 59.63 (4C) 106.36 (1C) 112.54 (2C) 121.99 (2C) 125.20 (2C) 126.13 (2C) 129.34 (2C) 132.67 (2C) 137.15 (2) 159.77 (2C) 180.38 (2C) 180.35 (1C). IR ν_max_ (cm^−1^) 3422, 2930, 1616, 1597, 1511, 1487, 1463, 1397, 1316, 1246, 1163, 1107, 996, 983, 873, 752, 689. HRMS: Calcd for C_27_H_33_N_2_O_4_Pd [M + H]^+^: 555.1475, found: 555.1489.

### 3.3. Cell Cultivation and Cytotoxicity

Biological tests were done on HT-29 and DLD-1 human colorectal cancer cell lines obtained from the European Collection of Cell Cultures (ECACC, Salisbury, United Kingdom). HT-29 was cultured in McCoy’s 5A Modified Medium and DLD-1 in RPMI-1640 cell culture media, both supplemented with Fetal Calf Serum 10%, l-glutamine and penicillin-streptomycin (Sigma-Aldrich, St. Louis, MO, USA). CSC human primary hepatocarcinoma cells and LIV normal hepatic cells were isolated and cultivated as described earlier [48]; they were grown in Dulbecco’s Modified Eagle Medium (with 4500 mg glucose/L) and Nutrient Mixture F-12 in 1:1 ratio, supplemented with Fetal Calf Serum (FCS) 10%, l-glutamine, penicillin-streptomycin, sodium pyruvate, β-mercaptoethanol and non-essential aminoacids (1% all the supplements). Normal human CD34^+^ lymphocytes, obtained by magnetic separation from whole population using CD34 microbeads and MACS magnetic separation system (from Miltenyi Biotech, Bergisch Gladbach, Germany) were cultivated in RPMI-1640 cell culture media, supplemented with FCS 10%, l-glutamine and penicillin-streptomycin. In the case of colon and hepatic cell lines, the experiments were done at 70–80% cell confluence, while for lymphocytes the cells were used immediately after the separation; the tests were confirmed by at least three independent measurements.

Cytotoxicity evaluations were done with 3-(4,5-dimethylthiazol-2-yl)-2,5-diphenyltetrazolium bromide (MTT) test (Sigma-Aldrich) for HT-29, DLD-1, CSC and LIV cells. The 3-(4,5-dimethylthiazol-2-yl)-5-(3-carboxymethoxyphenyl)-2-(4-sulfophenyl)-2*H*-tetrazolium/phenazine methosulfate (MTS/PMS) (Promega Corporation, Madison, WI, USA) colorimetric cell proliferation assay was done on normal human lymphocytes, following a protocol established previously [49]. Briefly, cells were seeded in triplicate in 96-well flat-bottom plates, at a cell population density of 2 × 10^4^ and 15 × 10^3^ for MTT and MTS/PMS assays, respectively. After 24 h, variable concentrations of the ligand and its palladium complexes (0.001–500 μg/mL) were added and the cells were incubated for additional 24 h. The half maximal inhibitory concentration (IC_50_) values were calculated as the concentrations corresponding to a 50% reduction of the cellular growth.

### 3.4. Quantitative Determination of Human Stem Cell Factor/c-Kit Ligand with ELISA

The in vitro SCF secreted by the colon carcinoma cell lines, hepatic cell lines and by the human normal lymphocytes was measured with ELISA quantitative sandwich enzyme immunoassay technique from the supernatants of the cells using the Quantikine human SCF Immunoassay (R&D Systems Europe Ltd., Abingdon, UK), and it was carried out according to the manufacturer’s procedures. Briefly, the cells were seeded in triplicate in 24-well plates at a cell population density of 4 × 10^5^ cells/mL. After 24 h of incubation, cells were treated with the free ligand and with Pd(II) complexes **1** and **2** at their IC_50_ concentrations and the supernatants were collected. After removal of the treatment, fresh media was added to the cells, and another harvesting of supernatant was performed for 24 h. The samples were centrifuged, aliquot and stored at ≤−80 °C. Monoclonal antibodies specific for SCF pre-coated onto microplates were used as capture antibodies. Standards and samples were pipetted into the wells and the SCF protein bound to the immobilized antibody. After washing away the unbound substances, an enzyme-linked polyclonal antibody specific for SCF was added to the wells. After removing the unbound antibody-enzyme reagent, a substrate solution was added to the wells and color developed proportionally with the amount of the SCF bound in the initial step. The color development was stopped and the optical densities were recorded using the ELISA microplate reader (Tecan Sunrise, Grödig, Austria) at 450 nm, with the correction wavelength set at 540 nm. The concentrations of the SCF were determined using the standard curve obtained from recombinant SCF, and were expressed as pg/mL.

### 3.5. Immunofluorescent Staining and Flow-Cytometry Analysis

Allophycocyanin (APC) labeled CD133/1 (AC133 clone, from Miltenyi Biotech, Bergisch Gladbach, Germany) anti-human monoclonal antibodies were used in order to identify CD133, which is a 5-transmembrane cell surface antigen with a molecular weight of 117 kDa [50]. Identification and analysis of CD133/1(AC133)^+^ cells was performed by flow-cytometry (FACS Canto II flow cytometer, BD Biosciences, San Jose, CA, USA) using the 633-nm, red, 17-mW HeNe excitation laser. The 660/20 filter was used to detect APC, the fluorescent marker of CD133.

The labeling of positive tumor cells was described before [51]; briefly, the cells were seeded in triplicate in 24-well plates at a cell population density of 4 × 10^5^ cells/mL. They were treated after 24 h of incubation with the free ligand and with complexes **1** and **2** using the concentrations corresponding to their IC_50_ values. Cells were removed from the plate by trypsinization, washed with CellWash buffer (from BD Biosciences), and up to 10^6^ cells were re-suspended in 80 μL phosphate buffered saline solution (PBS) with 0.5% FCS. 10 μL of FcR blocking reagent (from Miltenyi Biotec) was added in order to increase the specificity of the antibody and thereby to improve the purity of target cells. Afterward CD133/1 (AC133) antibody was added (10 μL) and the samples were incubated for 10 min in the dark, at 2–8 °C. Finally, cells were washed and re-suspended in 500 μL buffer for flow-cytometry analysis. Biostatistics was performed using the GraphPad Prism5 software (Graph Pad, San Diego, CA, USA).

## 4. Conclusions

The activity in vitro of studied compounds, points towards their applicability in living organisms as well. The cytotoxicity of complex **1** was prominent and the fact that complex **1** downregulates the CD133 expression in cancer cells along with the strong reduction of the SCF secretion in two aggressive tumor cell lines suggests a potential applicability as prodrug. Complex **2** activity against the tumor cells is weaker than complex **1**. On the other hand, compound **2** shows the best selectivity towards hematopoietic progenitor CD34 positive stem cells and reduces to a lesser extent the SCF expression in normal cells. The free ligand although less cytotoxic, inhibits the CD133 expression in hepatocarcinoma cells and in HT-29 colon carcinoma and generally it does not decrease the soluble SCF concentration. The difference of biological activity between complexes **1** and **2** may appear from the diamminepalladium moiety. As far as for the tetramethylcyclohexane-1,2-diamine the chair structure is energetically favored, the steric hindrance is not an impediment. In the case of dimethylpiperazine found in complex **2**, the predicted boat conformation may obstruct the biological activity. The novel Palladium(II) complexes **1** and **2** with 1,7-bis(2-methoxyphenyl)hepta-1,6-diene-3,5-dione ligand, obtained by biology-oriented synthetic methods, exhibit enhanced biologic activity against human colon and hepatic tumor cells by targeting the stem-like cancer cell populations. This indicates that the complexes metal-curcumin-like ligands are potential compounds able to arrest the tumor growth and to reduce the cell migration.

## Figures and Tables

**Table 1:** Cytotoxicity of the tested compounds (IC_50_, μM), calculated from the dose-response nonlinear regression (log inhibitor vs. normalized response, in the 95% confidence interval).

Cell Type	Curcumin Analogue		Complex 1		Complex 2	
	IC_50_ (μM)	SEM	IC_50_ (μM)	SEM	IC_50_ (μM)	SEM
**HT-29**	>500	-----	9.78	0.3	69.77	5.6
**DLD-1**	123.3	8.5	5.14	0.4	42.79	4.7
**CSC**	34.5	4.6	4.41	0.3	28.18	0.4
**LIV**	427.2	51.5	13.01	1.3	39.17	0.5
**LyCD34^+^**	223.8	20.3	10.65	1.5	129.00	14.5

**Table 2:** CD133 level variations in human colon and hepatic cells following the treatment; data provided by BD FacsDiva 6.1 software from 10,000 individual measurements in duplicate, median values and coefficient of variation (CV) calculated with GraphPad Prism software.

Cell Type	CD133 Positive Cells/10,000 Total Cells							
	Untreated Cells		Curcumin Analogue		Complex 1		Complex 2	
	Mean	CV	Mean	CV	Mean	CV	Mean	CV
**HT-29**	2172	0.039	1946	0.029	1404	0.041	2120	0.015
**DLD-1**	12	0.018	39	0.072	58	0.057	41	0.098
**CSC**	113	0.015	118	0.071	104	0.020	150	0.037
**LIV**	28	0.030	47	0.013	142	0.049	76	0.074
**LyCD34**	100	0.028	182	0.062	116	0.085	47	0.020

## Supplementary Materials

The supplementary materials are available online.
