# Supplementary material for: Novel Palladium(II) Complexes that Influence Prominin-1/CD133 Expression and Stem Cell Factor Release in Tumor Cells
Source: Molecules. 2017 Mar 30;22(4):561. doi: 10.3390/molecules22040561 (PMC6154565; doi:10.3390/molecules22040561)
Supplement: Supplementary file 1 [file molecules-22-00561-s001.pdf]

Supplementary materials

Novel Palladium(II) Complexes that Influence Prominin-1/CD133 Expression and Stem Cell Factor Release in Tumor Cells

Eva Fischer-Fodor, Roman Mikláš, Lucia Rišiaňová, Mihai Cenariu, Ioana-Georgeta Grosu, Piroska Virag, Maria Perde Schrepler, Ciprian Tomuleasa, Ioana Berindan Neagoe, Ferdinand Devínsky, Natalia Miklášová

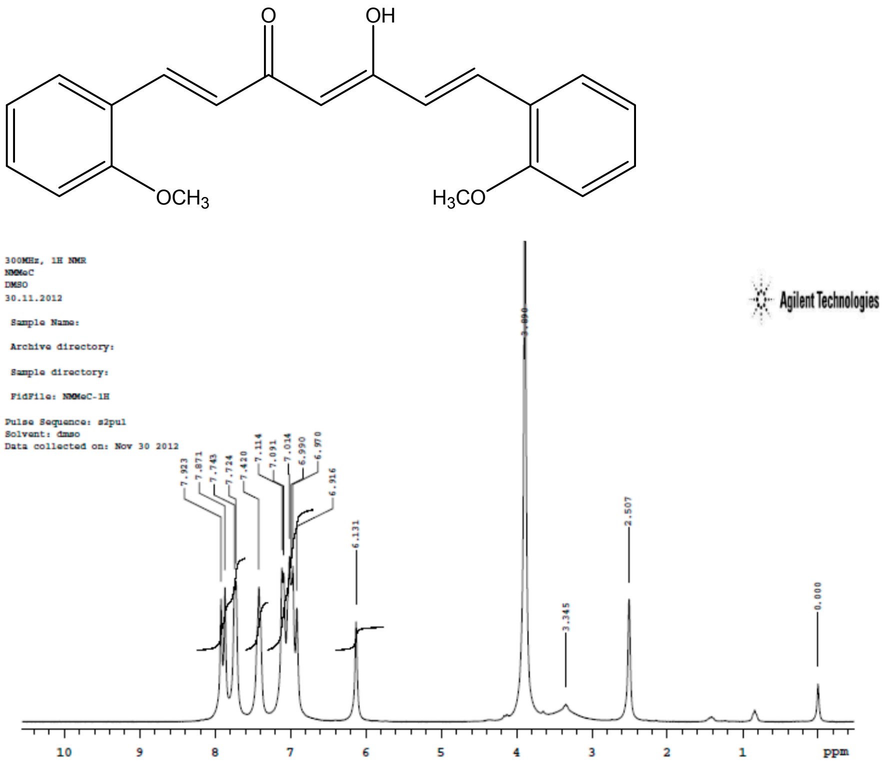

Figure S1. ^{1}H NMR spectrum of 1,7-bis(2-methoxyphenyl)hepta-1,6-diene-3,5-dione

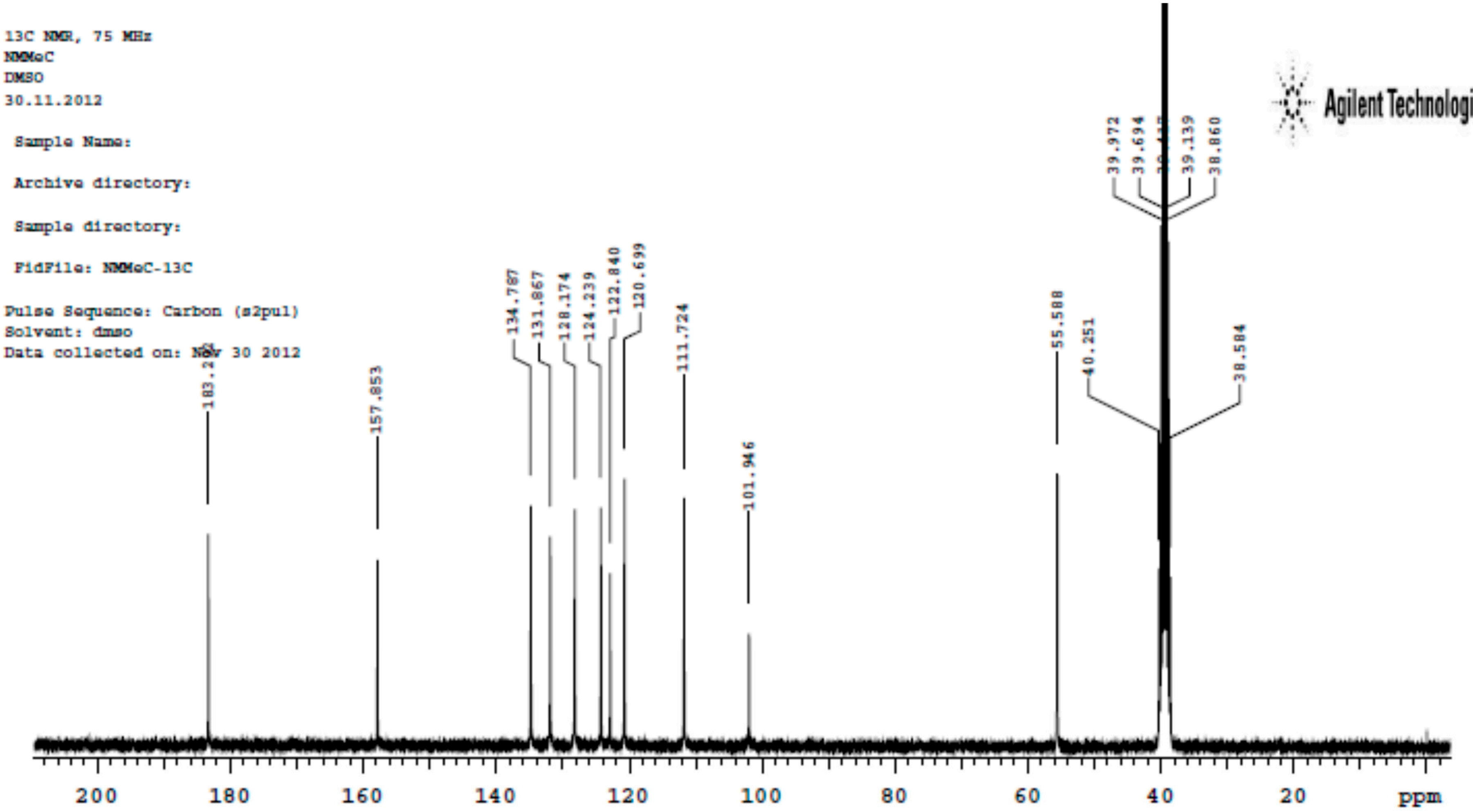

Figure S2. ^{13}C NMR spectrum of 1,7-bis(2-methoxyphenyl)hepta-1,6-diene-3,5-dione

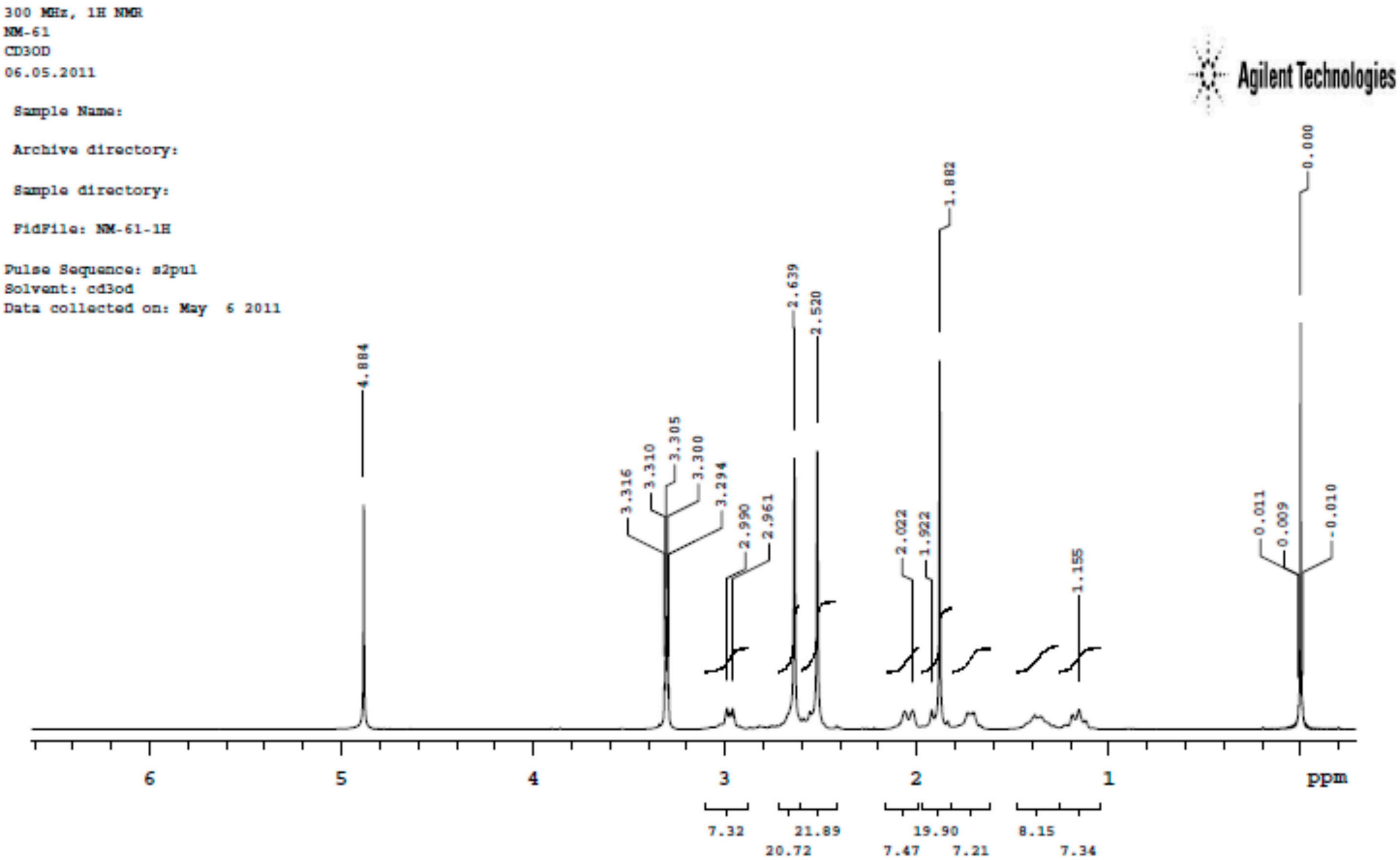

Figure S3. ^{1}H NMR spectrum of palladium complex containing *N,N,N′,N′*-tetramethylcyclohexane-1,2-diamine [($C_{10}H_{22}N_2$)Pd(OAc)$_2$]

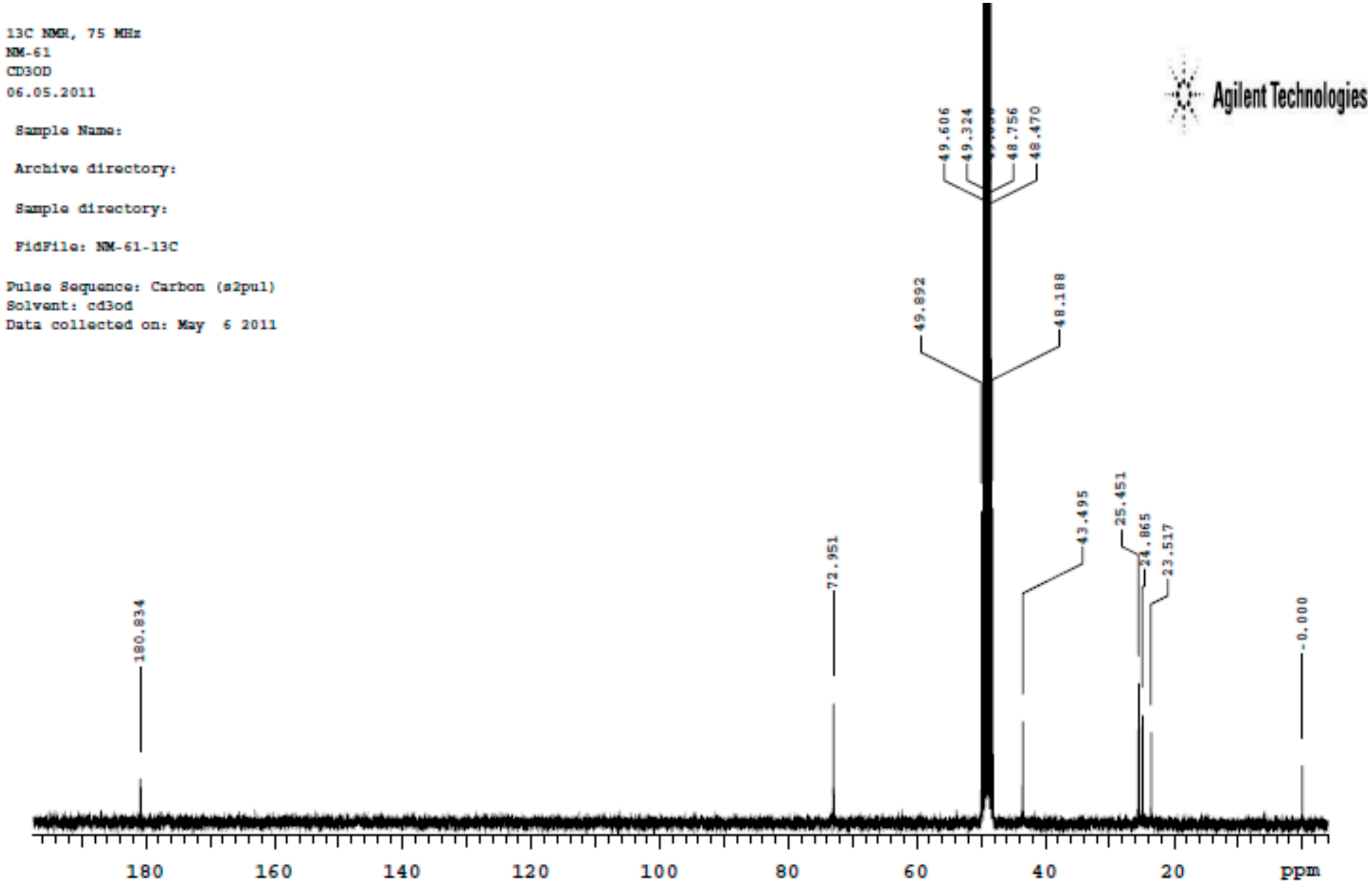

Figure S4. ^{13}C NMR spectrum of palladium complex containing *N,N,N′,N′*-tetramethylcyclohexane-1,2-diamine [($C_{10}H_{22}N_2$)Pd(OAc)$_2$]

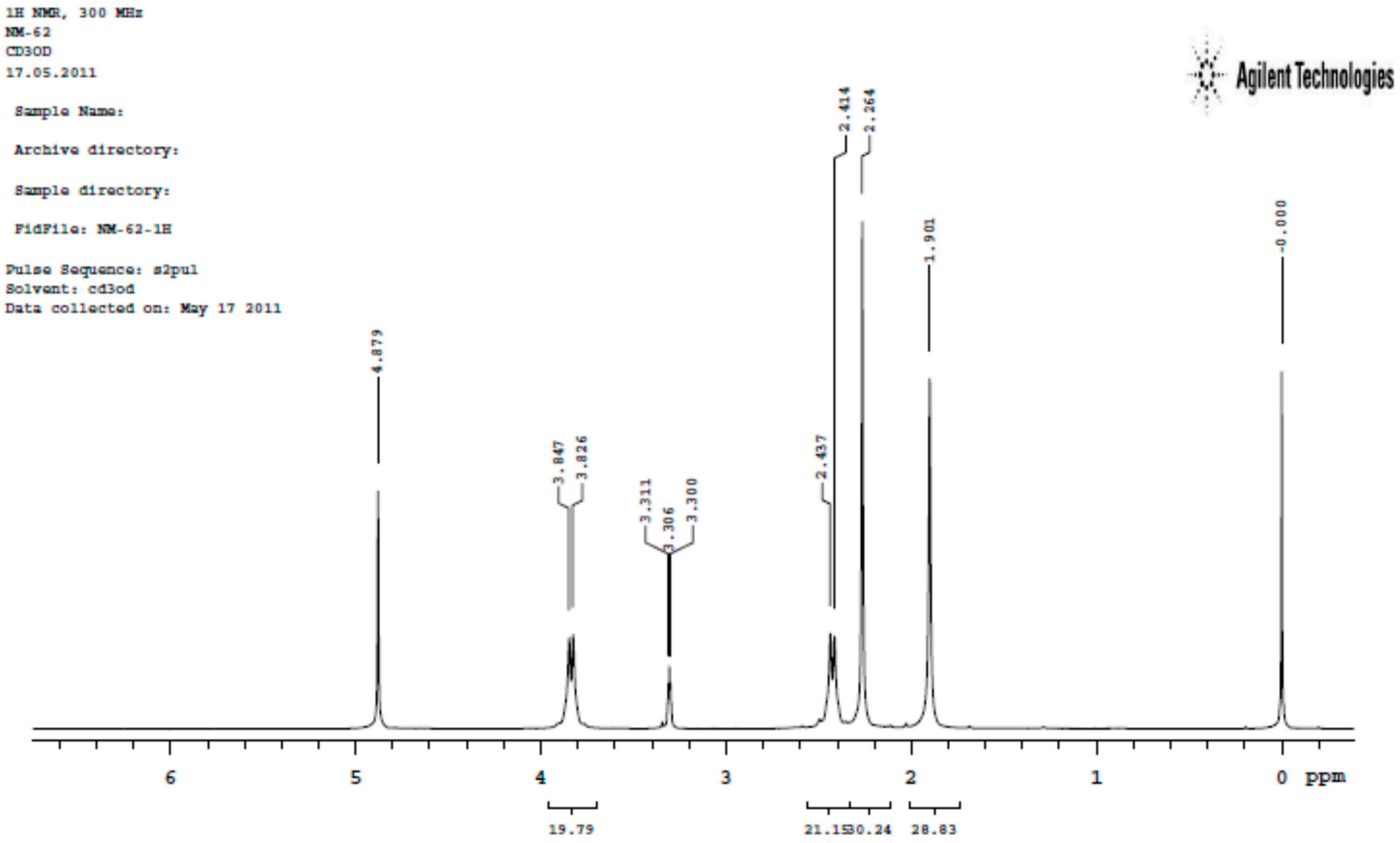

Figure S5. ^{1}H NMR spectrum of palladium complex containing 1,4-dimethylpiperazine [($C_6H_{14}N_2$)Pd(OAc)$_2$]

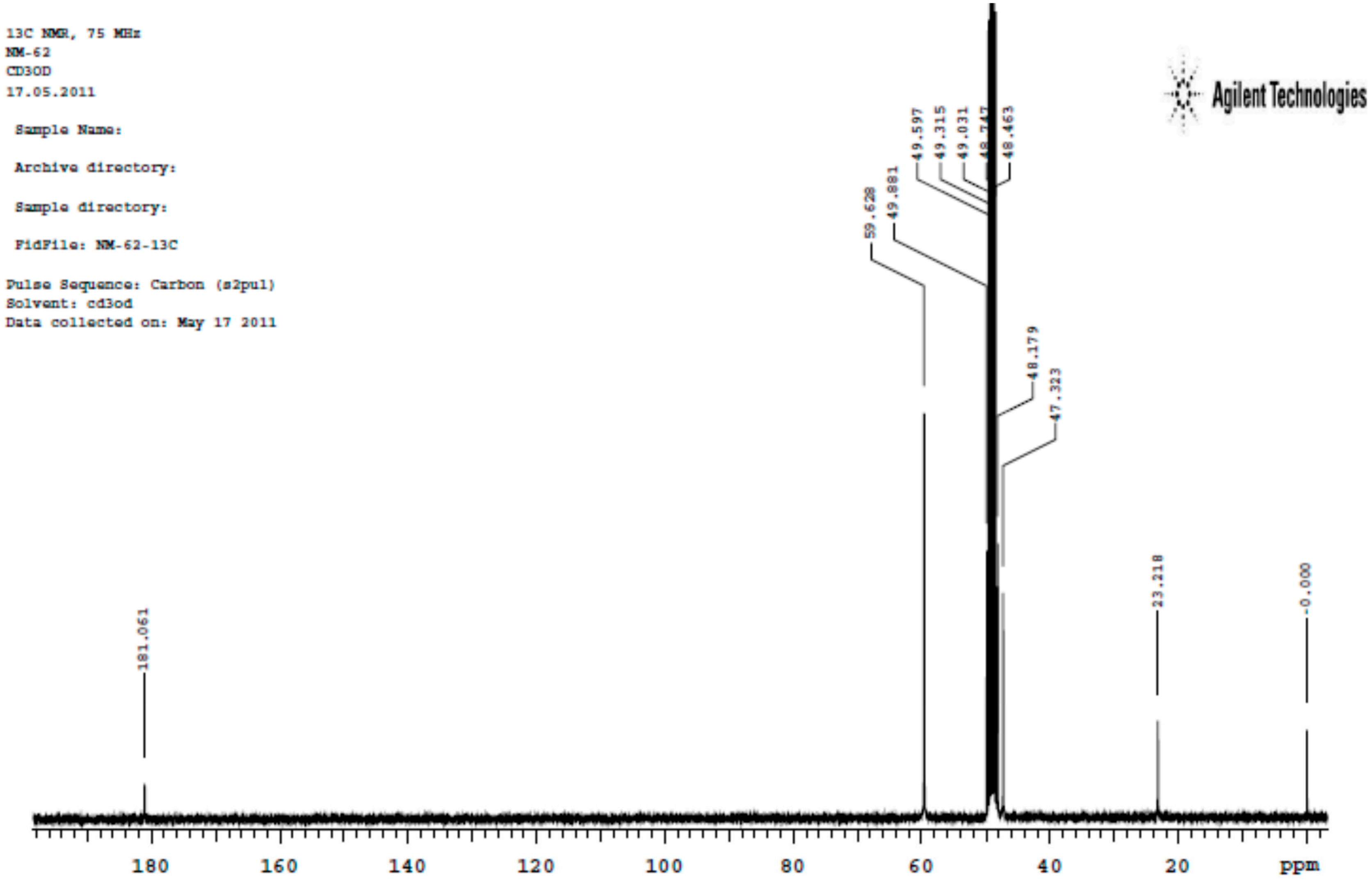

Figure S6. [13] C NMR spectrum of palladium complex containing 1,4-dimethylpiperazine [$(C_6H_{14}N_2)Pd(OAc)_2$]

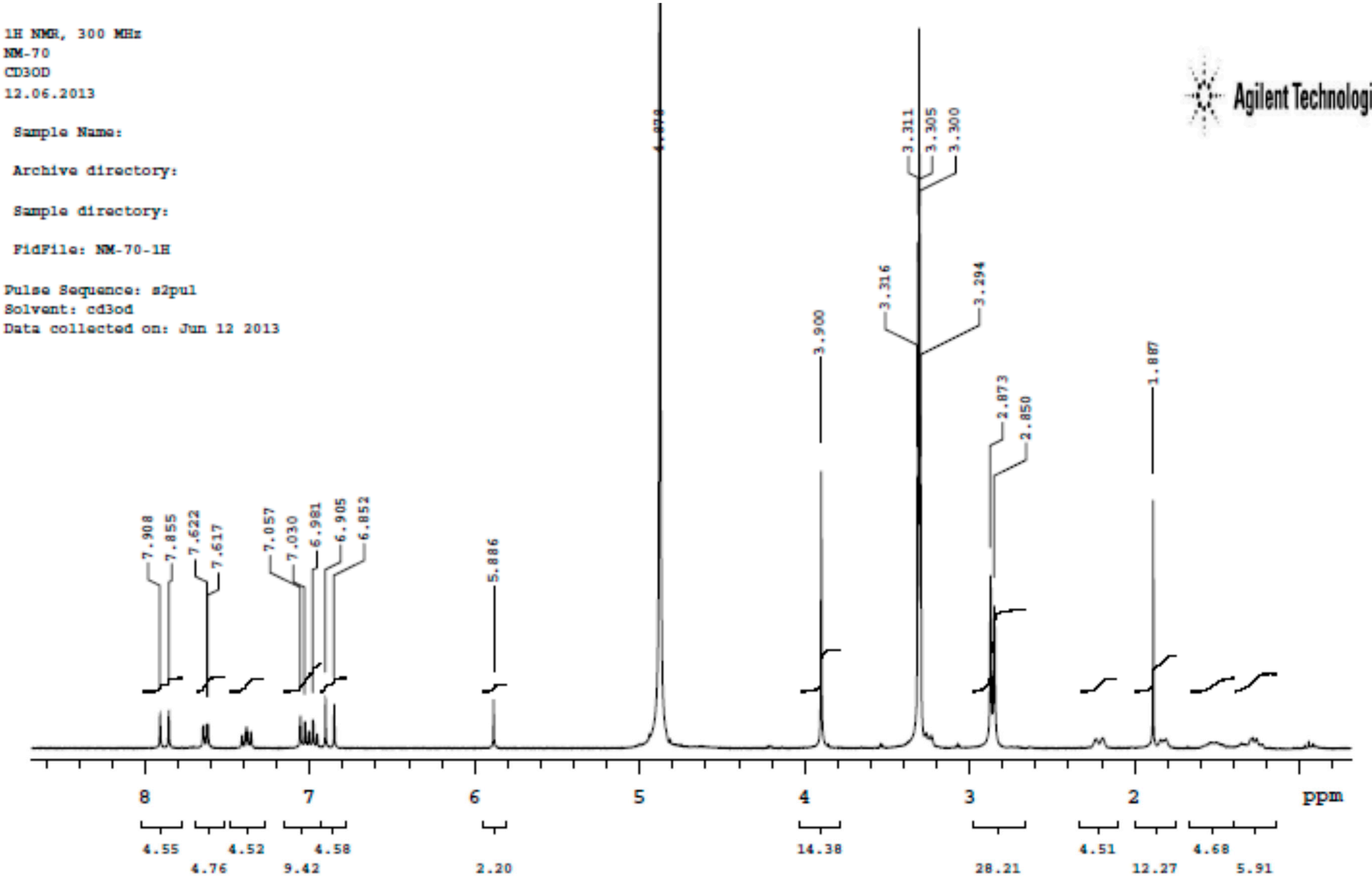

Figure S7. ^{1}H NMR spectrum of Pd(II) complex **1**

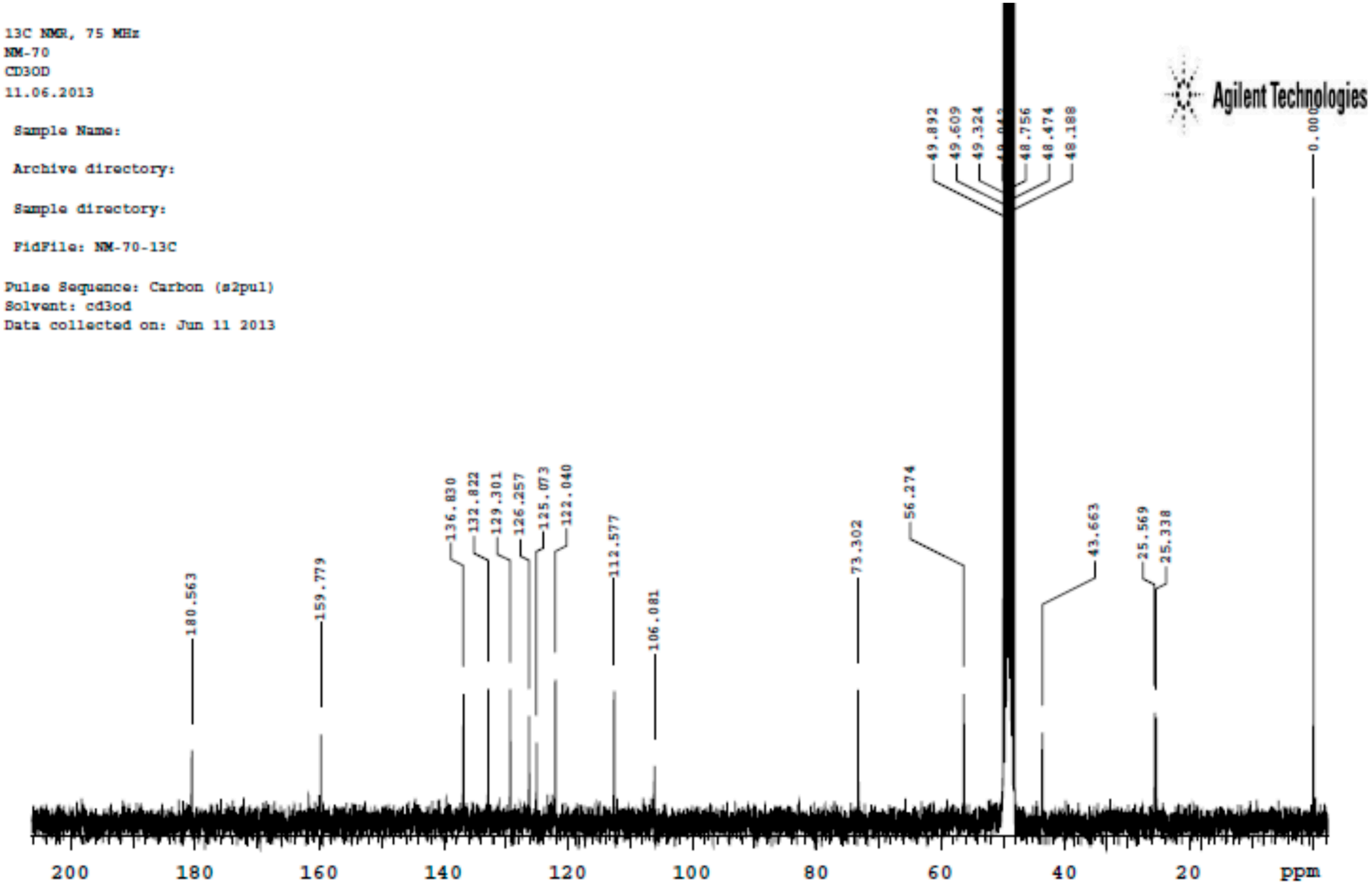

Figure S8. ^{13}C NMR spectrum of Pd(II) complex **1**

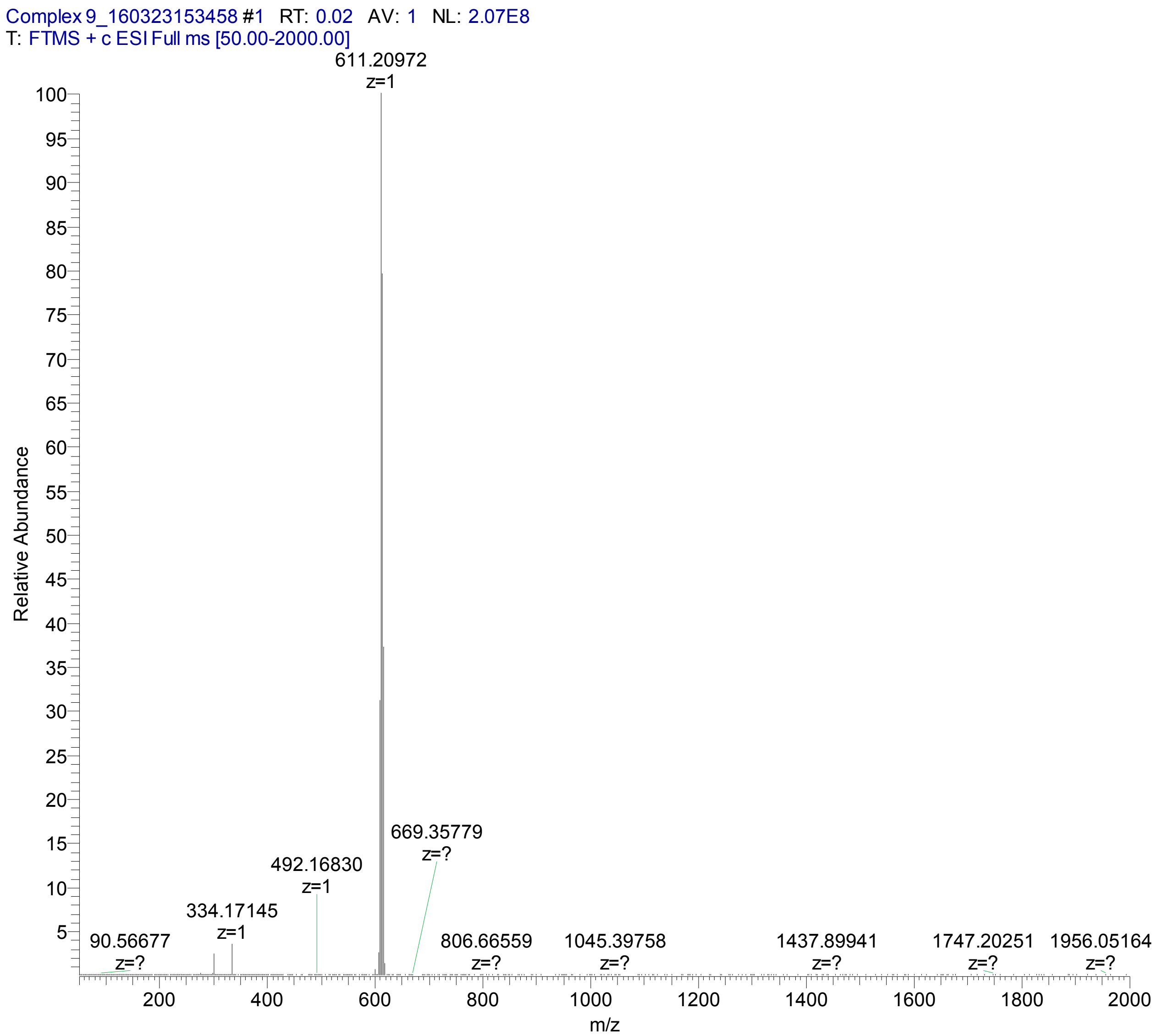

Figure S9. HR-MS spectrum of Pd(II) complex **1**

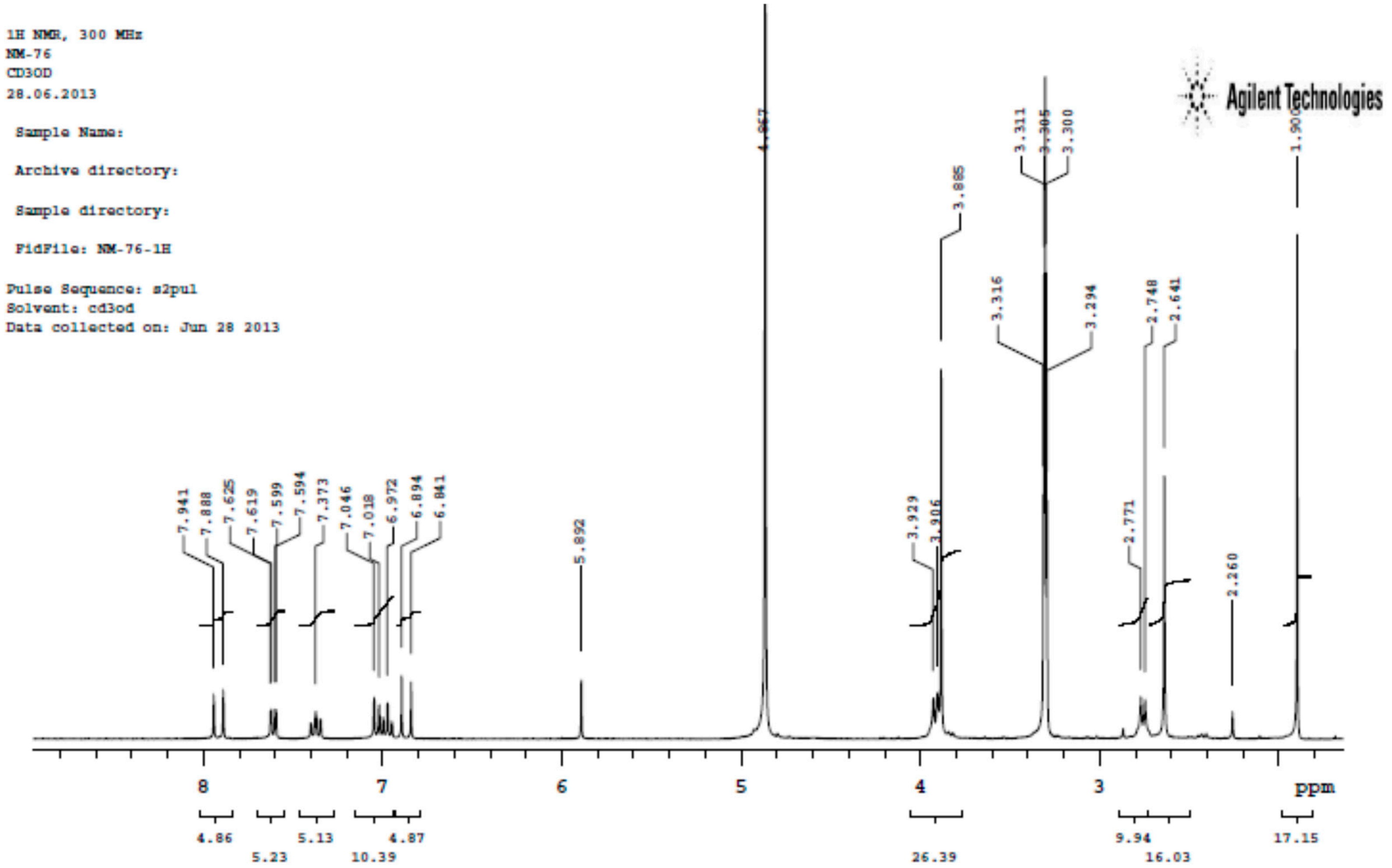

Figure S10. ^{1}H NMR spectrum of Pd(II) complex **2**

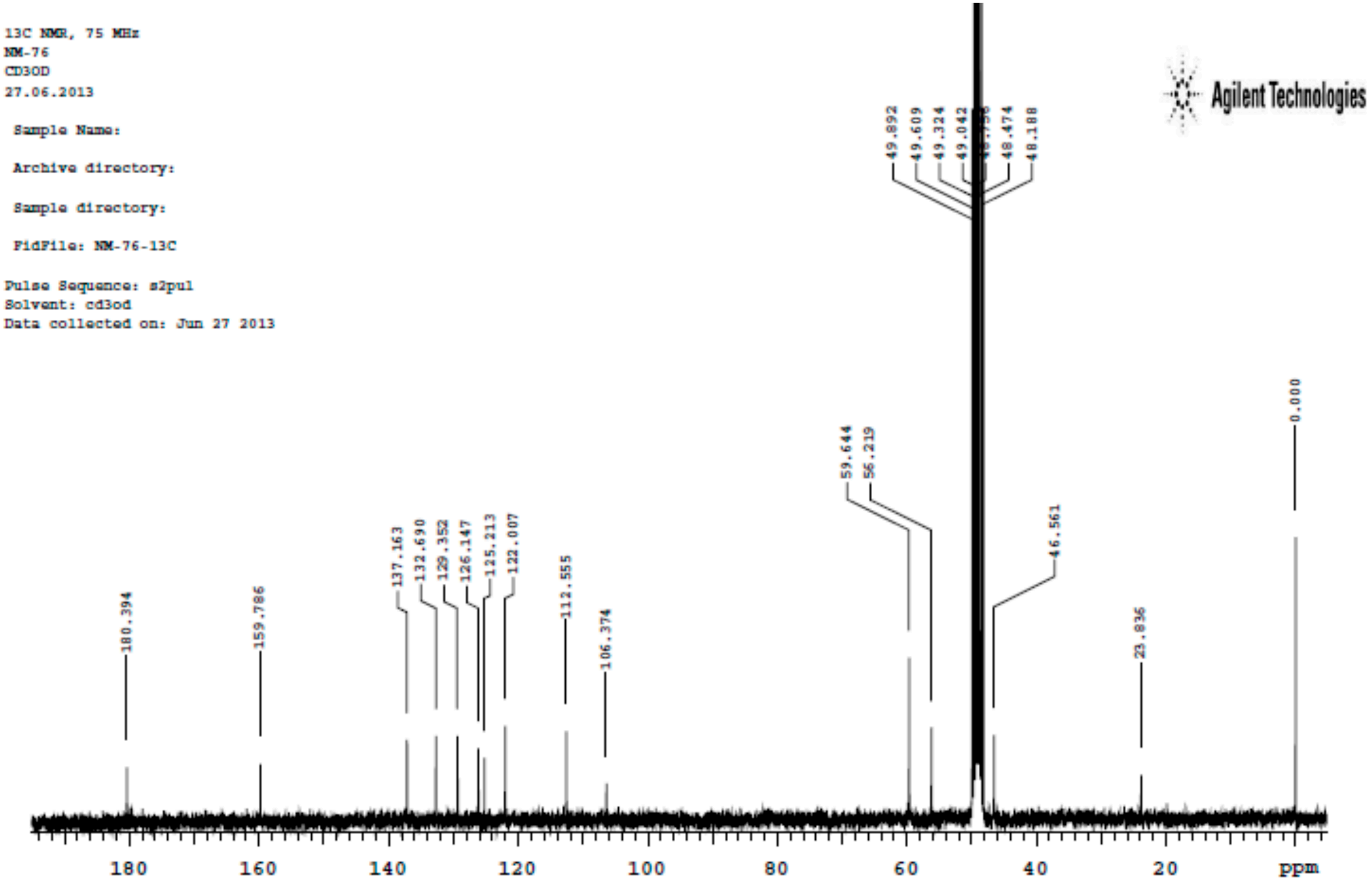

Figure S11. ^{13}C NMR spectrum of Pd(II) complex **2**

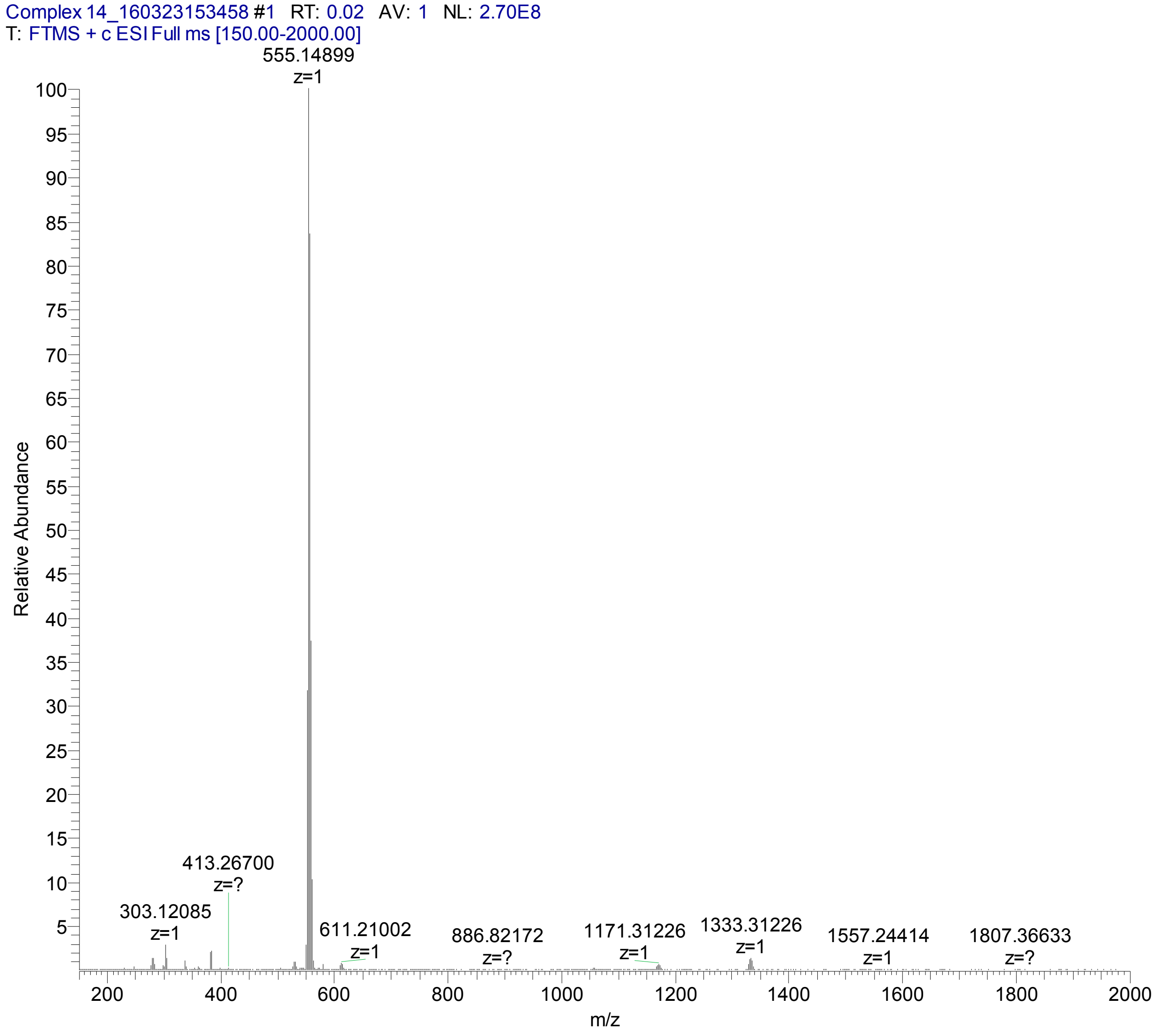

Figure S12: HR-MS spectrum of Pd(II) complex **2**
